# An sEMG-Controlled 3D Game for Rehabilitation Therapies: Real-Time Time Hand Gesture Recognition Using Deep Learning Techniques

**DOI:** 10.3390/s20226451

**Published:** 2020-11-12

**Authors:** Nadia Nasri, Sergio Orts-Escolano, Miguel Cazorla

**Affiliations:** University Institute for Computer Research, University of Alicante, P.O. Box 99, 03080 Alicante, Spain; sorts@ua.es (S.O.-E.); miguel.cazorla@ua.es (M.C.)

**Keywords:** electromyography sensor, deep learning, hand gesture recognition, virtual reality, rehabilitation

## Abstract

In recent years the advances in Artificial Intelligence (AI) have been seen to play an important role in human well-being, in particular enabling novel forms of human-computer interaction for people with a disability. In this paper, we propose a sEMG-controlled 3D game that leverages a deep learning-based architecture for real-time gesture recognition. The 3D game experience developed in the study is focused on rehabilitation exercises, allowing individuals with certain disabilities to use low-cost sEMG sensors to control the game experience. For this purpose, we acquired a novel dataset of seven gestures using the Myo armband device, which we utilized to train the proposed deep learning model. The signals captured were used as an input of a Conv-GRU architecture to classify the gestures. Further, we ran a live system with the participation of different individuals and analyzed the neural network’s classification for hand gestures. Finally, we also evaluated our system, testing it for 20 rounds with new participants and analyzed its results in a user study.

## 1. Introduction

According to the World Health Organization (WHO) (https://www.who.int/disabilities/world_report/2011/report/en/), about 15% of the world’s population lives with some form of disability, of whom many suffer from musculoskeletal or nervous system impairments. Rehabilitation processes may enable a patient to restore their functional health. Rehabilitation refers to a set of interventions needed of repeating exercises to strengthen the damaged body part. A rehabilitation process can be exhausting, painful and difficult and the exercises must be performed in a specific way to allow patients to maximize their chances of recovery and regain maximum self-sufficiency [1,2].

In recent years, there has been an increasing interest in rehabilitation methods using advances in technology with the aim of enhancing patient comfort. Certain points need to be considered for rehabilitation exercises to be more efficient. The process should be user friendly and lead the patients to feel motivated to engage in the exercise and enjoy the experience [3,4]. Applying competitive stimuli and scoring mechanisms to the exercises (according to the correct movements) can help fulfill specific objectives during the rehabilitative process. Moreover, the rehabilitation exercises must be customizable depending on the therapist’s indications and the therapist should be able to apply any suitable adjustments in the line with the patient’s conditions [5,6].

Interactive systems, which support interaction between humans and machines, such as Augmented Reality (AR) and Virtual Reality (VR), have increased the possibilities of helping the rehabilitation process. As progress in rehabilitation needs time, much effort has focused on using these interactive systems to reduce the need for a patient’s regular attendance at rehabilitation clinics and to provide the opportunity to perform a set of physical exercises in a home-based environment [7,8,9].

However, a major significant drawback of these kinds of applications is that they increase the likelihood of the exercises being performed in a casual and incorrect manner. If the therapist simply relies on the final results of each section and fails to control the complete process, the effects of the exercise may be inadequate [1].

Virtual Reality (VR) forms part of the most recent generation of computer gaming. This technology provides the opportunity to experience action games from a first-person perspective in a simulated world. The newest rehabilitation methods leverage Machine Learning techniques and VR systems. These classifiers receive labeled data from sensors and/or cameras for each movement performed by healthy subjects to learn its high-level features [10,11,12,13].

Since electromyography (EMG) sensors can record the electrical activity produced by muscles, many studies have focused on the effectiveness of these sensors in the rehabilitation process [14,15,16,17]. A surface EMG (sEMG) is a useful non-intrusive sensor to facilitate the study of muscle activity from skin. In this work, we evaluate sEMG data for gesture recognition tasks during the rehabilitation process by means of implementing of Deep Learning systems.

This paper presents a dataset with 7 static hand gestures acquired via sEMG from 15 individuals. The novel dataset was tested by a proposed neural network architecture. In addition this paper introduces a rehabilitation game controlled by seven dissimilar hand gestures for patients who require a recovery process due to their experiencing limitations in everyday functioning caused by aging or a health condition. The main contributions of this paper are:

- A novel dataset acquired from 15 subjects with 7 dissimilar hand gestures.
- A deep learning-based method for hand gesture recognition via sEMG signals.
- A live 3D game for rehabilitation, that leverages AI (hand gesture recognition), to create a compelling experience for the user (rich visual stimuli).

The rest of the paper is organized as follows: Section 2 reviews the state of the art related to existing hand gesture datasets recorded using EMG and sEMG sensors. Moreover, we review existing learning-based approaches and their use in rehabilitation processes. Next, in Section 3, the details of the 3D game and the capture device we used to compose the dataset are described. In addition, this section describes the system and architecture of the neural network used to train the hand gesture recognition dataset and the EMG-based rehabilitation system. Section 4 presents the results obtained and describes the experimental process. In Section 5 we describe the experience of users and their opinions. Section 6 presents the main conclusions of our work and suggests some future research lines.

## 2. Related Work

Every year, some 800,000 people in the United States suffer a stroke and approximately two-thirds of these individuals survive and require rehabilitation. The goal of rehabilitation is to relearn skills that are suddenly lost after a stroke to improve the patients’ level of independence and help them return to their former social activities. Based on the literature, Post-Stroke Depression (PSD) is the most common neuropsychiatric consequence after stroke onset [18] that should be considered during the rehabilitation. The presence of PSD is correlated with failure to return to previous social activities and can have negative effects on the rehabilitation process [19]. Recent trends in PSD have led to a proliferation of studies suggesting rehabilitation exercises should use a reward system in order to motivate patients to to engage more completely with the process and to help avoid depression [20].

The rapid development of artificial intelligence has led to emerging areas of research in rehabilitation methods. One of the most auspicious treatments is VR, which can provide visual stimuli to assist in enhancing locomotor system progress. Its effectiveness in patients with different conditions has been studied [21,22,23].

The efficiency and effectiveness of wearable sensors have been studied in various rehabilitation contexts [24,25,26,27]. Electromyography sensors are one of the most widely used type of wearable sensors in rehabilitation tasks; they, enable the professional in charge to study and analyze the muscular activities of patients during the process [17,28,29,30].

Moreover, the function of electromyography signal in some clinical applications, human computer interaction, muscle computer interface and interactive computer gaming is considerable [31,32].

The role of muscular activity signals captured by EMG sensors has been noteworthy in the field of hand gesture recognition [33,34,35]. Several public datasets exist, which have been acquired by high-density surface electromyography (HD-sEMG) or regular sEMG electrodes, such as CapgMyo [36], csl-hdemg [37], or NinaPro (Non Invasive Adaptive Hand Prosthetics) [38,39]. The mentioned datasets have been recorded by high frequency electrodes, provide a wide variety of dynamic hand gestures in the associated studies. In CapgMyo (includes 8 hand gestures) and csl-hdemg (includes 27 finger gestures), datasets were recorded by a large number of HD-sEMG ( 128 and 192 sensors respectively) with a high sampling rate using dense arrays of individual electrodes, in order to obtain information from the muscles [36,37]. Datasets which are acquired by HD-sEMG signals are not appropriate under our framework in this research and only NinaPro databases contain regular sEMG electrodes in their recording process. The NinaPro database was recorded from a sample of 78 participants (in different phases) and 52 different gestures. In this project, 10–12 electrodes (Otto Bock 13-E200 (Ontario, Canada), Delsys Trigno (Natick, MA, USA)) were placed on forearms, plus one CyberGlove II with 22 sensors to capture 3D hand poses [38]. In the continuation the 5th Ninapro database is recorded from 10 subjects performing 52 dynamic hand movements. The database has been recorded via 38 sensors (two Myo armbands + Cyberglove 2) and includes three axis accelerometer information. All movements in the dataset are dynamic and it does not consist of static gestures [39]. Because of the multiplicity of costly sensors and dynamic hand movements, we decided to work with our previous dataset and extend it with a more static gesture.

However in the present work, we decided to use a sub-dataset of the dataset acquired in our previous work, which was recorded from hand gestures using low-cost sensors. Our dataset consisted of static hand gestures and we decided to study the behavior of a system with a smaller training group than in previous works [40,41].

Debate continues about the best techniques for data extraction of EMG signals [42], although deep learning techniques are the subjects of great interest as novel methods of feature extraction for gesture classification [43,44,45]. There are several studies on EMG signals captured by the Myo armband with low frequency (200 Hz) and applied neural network models for dynamic gesture recognition tasks [39,40,43]. In recent years, there have been an number of studies on the capability of this user-friendly armband in rehabilitation issues [46,47,48].

The original software (proprietary) from the Myo armband contains only five hand gestures and its default system has a considerable error rate in predicting correct hand gestures in real time. Hence, in the present work, we decided to expand the number of gestures through the use of deep learning techniques. In our previous work [40], we examined the efficiency of gated recurrent unit (GRU) architecture [49] on raw sEMG signals for six gestures and created an application for controlling domestic robots to help disabled people [41].

The results of recurrent neural networks (RNN) demonstrate that this type of architecture is a suitable choice in sequential problems [50,51], as well as for GRU in raw speech signals [52].

Convolutional GRU (ConvGRU) architecture is widely used for learning spatio-temporal features from videos [53,54,55]. Therefore, we decided to implement a ConvGRU-based neural network architecture. The proposed architecture takes raw sEMG signal as input and is able to estimate user hand gesture as output.

## 3. Sensor-Based Rehabilitation System

The rehabilitation system is divided into four phases: Database and Recording Hardware, Neural Network Architecture, Rehabilitation API and System Description.

### 3.1. Database and Recording Hardware

In our previous work, we created a dataset of six dissimilar static hand gestures acquired from 35 healthy subjects. Each gesture was recorded for 10 s by maintaining the hand gesture and moving arms in various directions. The dataset collected, which contains approximately 41,000 samples, was set as input to train a 3-GRU layer neural network. EMG signals were applied as raw data for the training system, which achieved an accuracy of 77.85% in a test examination with new participants [40].

The goal of this work was to add more gestures and a new neural network architecture to minimize the prediction time and maximize the system’s accuracy. Our previous study [40] found, the Myo armband was unable to distinguish more than 6–7 gestures accurately. Hence, in this work we randomly chose 15 subjects from the previous dataset, acquiring a further gesture (Neutral) from each one to study the results with a new proposed architecture. The additional gesture was recorded in the same condition as the previous gestures. The armband was placed at the height of the Radio-humeral joint, with participants being asked to maintain the requested hand gesture, move their arms in various directions and avoid completely bending the forearm at the elbow joint. The raw signals recorded from the EMG sensors were transmitted via Bluetooth (at 200 Hz) in eight channels for each samples.

**Figure 1:**
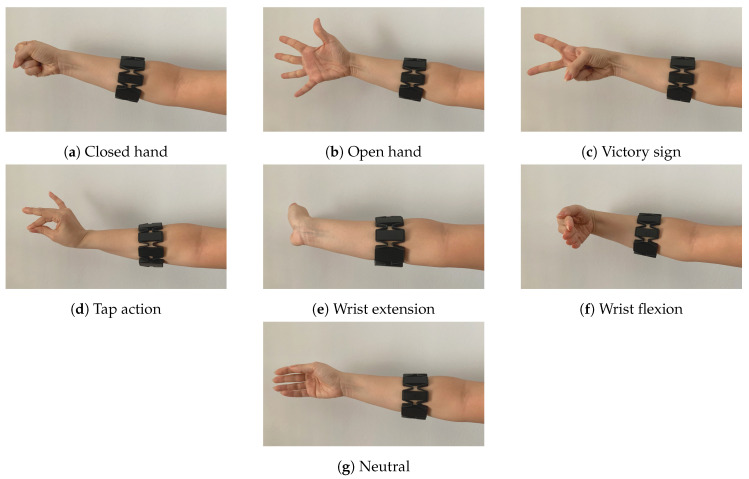
Hand gestures.

The new sub-dataset contains 18,500 samples from 15 healthy subjects for 7 gestures (open hand, closed hand, victory sign, tap, wrist flexion, wrist extension and neutral) (See Figure 1). The dataset is publicly available in our website (http://www.rovit.ua.es/dataset/emgs/).

### 3.2. Neural Network Architecture

A variety of methods are used to assess gestures; each has its particular advantages and drawbacks [56,57,58,59]. The most popular neural network for sEMG-based gesture recognition is the convolutional neural network (CNN), although attention has recently been increasing in Recurrent neural networks (RNN) for the same task. The results show the veracity of both architectures in sEMG-based gesture recognition. CNN-LSTM models have also been proposed for gesture recognition and extracting features from RGB videos [60]. However, in this work we propose a combination of CNN and GRU models to apply to sEMG signals.

Since the dataset was acquired as signals, we chose to work with one-dimensional, convolutional blocks for the proposed neural network. No filter and pre-processing process on network input was implemented.

The Myo signals are zero-centered by default and a normalization method was not necessary. With the aim of supervised learning, we implemented two methods for labeling our outputs. First, we used a one-hot encoded vector and introduced the correct gesture with the value of 1 in the intended index of the vector and incorrect gestures were given the value of 0. Second, we used soft label assignment for our supervised learning and gave an insignificant probability to the incorrect gestures (0.01) and high probability to the correct ones.

To improve the system’s accuracy, the window method was implemented as the data augmentation technique. We maintained the results of previous experiments and considered a sliding window with 188 as the look-back per sample and 20 as the offset in each window. In the proposed Conv-GRU architecture, we implemented two layers of Convolutional 1D with 64 feature detectors with a kernel size 3 applied to each feature detector. The next layer was a Max Pooling layer that slides a window of 3 across our data and this was followed by two Convolutional 1D layers with 128 filters and a kernel size 3. In addition, to avoid the possibility of over-fitting a dropout layer with 0.5 probability of neuron activation was considered. To extract temporal features, two GRU layers were added. Each GRU layer was composed of 150 units and to further reduce the likelihood of over-fitting and to enhance the model’s capabilities, a dropout layer with a rate of 0.5 was applied after each GRU layer.

**Figure 2:**
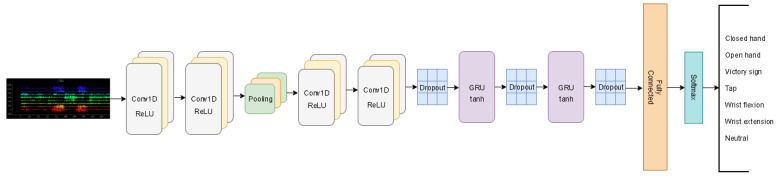
Proposed neural network architecture for hand gesture recognition.

For the final part of our proposed network, we used a fully connected (FC) layer with seven neurons matching the number of gestures. It uses the Softmax activation function to produce a probability distribution over the number of classes and control the actions of extreme values. The architecture described is shown in Figure 2.

All the parameters, such as number of layers, number of filters per layer, activation functions and dropout rates, were empirically chosen. In our experiments, we used an Adam optimizer with a constant learning rate of 0.0001 and a batch size of 500 for 300 epochs. For the loss function, the categorical-crossentropy function was chosen.

### 3.3. 3D Game Experience

**Figure 3:**
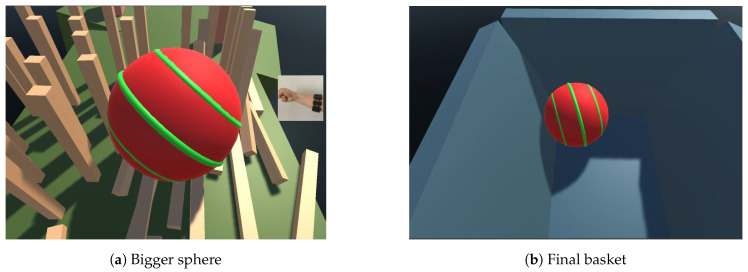
Three-dimensional game.

In the present work, we used Unity engine to create a three-dimensional game based on hand gestures. The game was created with a reward system to elicit positively-valenced emotions and so facilitate the rehabilitation process. In the proposed game, 5 hand gestures were selected to control a sphere to overcome different obstacles (by increasing speed, going backwards, jumping, making the sphere bigger or smaller) and then fall into a basket (See Figure 3). The final goal of the game is to overcome all the obstacles and drop the ball into a basket, which is designed to provide patients with the reward of successfully completing the task.

### 3.4. System Description

**Figure 4:**
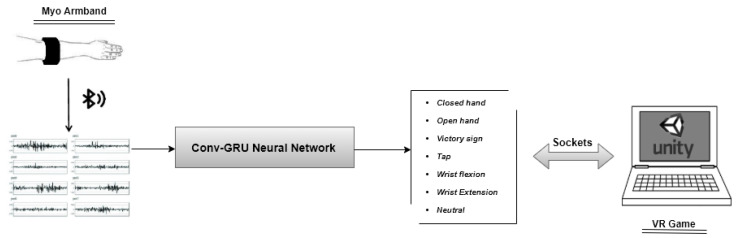
Proposed System.

In order to send information from the neural network to the 3D game, we used socket connections. The sEMG data was transmitted via Bluetooth to the Conv-GRU neural network. The proposed network analyzed the information and classified the gesture. The predicted gesture was sent to the game through socket connections. Unity received the class of gesture and ran the appropriate function introduced to perform the gesture. The system described is shown in Figure 4.

## 4. Experiments and Results

In order for the experiments and to yield realistic results, we divided 15 subjects into five different groups. To control for bias, a test examination was carried out in all groups. We implemented the leave-one-out Cross-Validation technique and the training process was conducted via GeForce GTX 1080Ti GPU. In each training section, four groups were used to train the system and the samples were split into 75% for training data and 25% for validation data. The validation data contained information from the same subjects as the training data, but at different moments, and the neural network did not see them during the training process.

**Figure 5:**
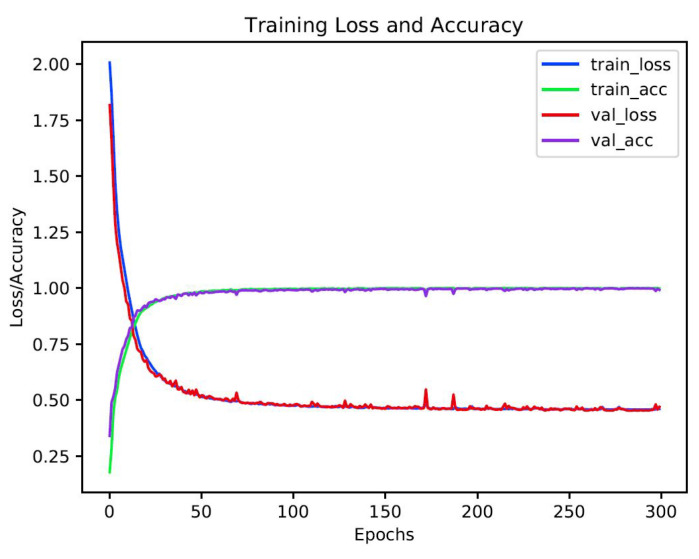
Loss and accuracy graph.

The training results after 300 epochs were convincing enough to do the test. In both implemented methods (one-hot vector and label smoothing) training accuracy was around 99.4–99.8%, followed by validation accuracy with an insignificant difference (See Figure 5).

**Figure 6:**
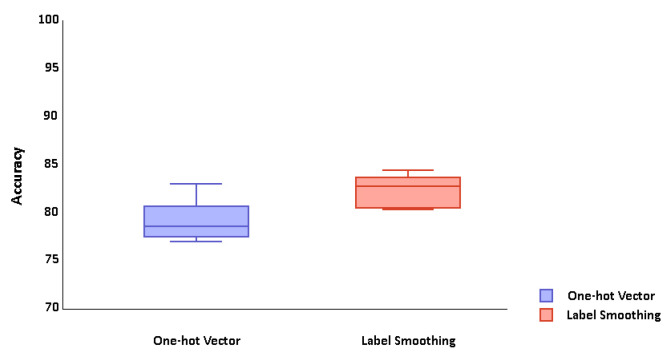
Cross validation graph.

The training process was repeated five times for both methods. Each time, one group was left out for testing and four groups were used to train the system. This process took 11 h. The average results of cross validation were considerably different. the average test accuracy for labeling by one-hot vector was 79.07%, and 82.15% for the label smoothing method (See Figure 6). As the significant difference in test results shows, the label smoothing method achieves greater accuracy. Consequently, we chose to train the neural network with this method and to implement the rehabilitation system.

**Figure 7:**
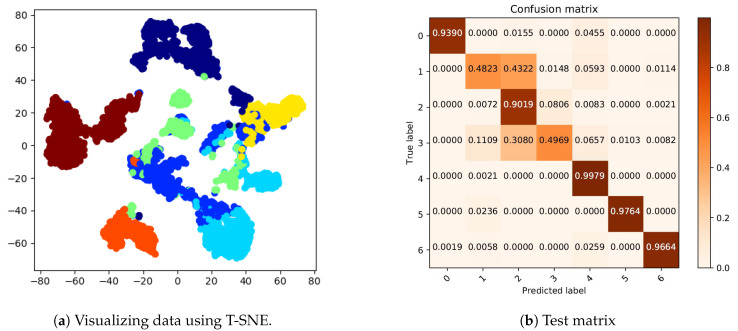
Confusion matrix.

For the output, we implemented a T-distributed Stochastic Neighbor Embedding (T-SNE) algorithm to reduce the dimension in 2D and visualize high-dimensional data by categorizing them by features [61]. This nonlinear dimensionality reduction allowed us to see how our proposed neural network was able to extract the characteristics of each hand gesture. Each cluster represents a hand gesture and the proposed neural network exhaustively classified sEMG signals (See Figure 7a).

In addition, to gather more details about the errors in hand gesture prediction, a confusion matrix was created (See Figure 7b).

As can be observed in the confusion matrix, the system could reasonably classify the closed hand, victory sign, wrist flexion, wrist extension and neutral gestures (there is a probability of a maximum ±0.05 error in each row or column, due to the rounding of numbers). However, the system had difficulties in distinguishing the open hand and tap gestures from the others and both had a significant conflict with the victory sign gesture.

Thereafter, we used five gestures (closed hand, tap, wrist flexion, wrist extension and neutral) to control the sphere in the game and left the other two gestures unused so as to give future users the opportunity of adding their desired actions to the game. The sphere should pass obstacles by changing the sphere size (making it bigger or smaller), jumping, accelerating and going backwards.

According to the system’s run time, each gesture was predicted in 39ms and we were able to send data to Unity through the socket. The socket connection was a two-way communication link between Python and Unity, running on a combination of an IP address and a port number. However 39 ms was insufficient time for unity to finish the movement before receiving another command. To prevent this polarization, a minimum delay (1 s) was applied between each sending of data from the neural network to the game.

## 5. User Study

With a view to having more realistic results, the system was tested live with 4 participants. All subjects were new and their information was unseen by the system during the training process. Participants were given a spoken explanation of the game and the gesture available for playing. Each subject was allowed two rounds to familiarize themselves with the route and obstacles. A round needs approximately one minute to be completed and each participant played five rounds. In addition, from one round to another, two minutes rest was considered (See Table 1).

Regarding the results of the live system and the users’ opinions, in the last round, the found it difficult to make the wrist flexion and wrist extension gestures perfectly as their hands were tired. However, the system was able to recognize the gestures correctly and the main reason for lost rounds was bad timing of gesture performance (each time the sphere hit the ground, the game was restarted). In spite of the results, competitiveness and focus on outcomes led the players to request more rounds.

## 6. Conclusions

This work proposed a new Conv-GRU architecture for the problem of gesture recognition. We also proposed a 3D game for use in rehabilitation processes. The new approach was trained and tested for seven static hand gestures. In the training process, the system achieved 99.8% in training accuracy and 99.48% in validation accuracy. The proposed network was tested with new subjects and obtained 82.15% accuracy in gesture recognition. An EMG-based system was designed to study its effectiveness and assistance in rehabilitation methods. Four new subjects took part in a live test and played a game based on controlling a sphere with hand gestures. Moreover regarding our experimental results, we demonstrated that the Conv-GRU network is sufficiently accurate to be used in gesture recognition systems and the proposed sensor based 3D game can be extended for application to rehabilitation techniques. To demonstrate our results, we have published a video in YouTube. (https://www.youtube.com/watch?v=ATNbnDGpMCk&feature=youtu.be).

For future work, we consider further hand gestures and harder levels for the rehabilitation 3D game. additionally, it is important to find means to increase the accuracy of the learning based system and also develop the system for inserting customized hand gestures for each patient in case of need.

## Figures and Tables

**Table 1:** Results for new subjects and their opinion.

	**Round1**	**Round2**	**Round3**	**Round4**	**Round5**	**Player’s Opinion**
**Subject 1**	Win	Win	Lose	Win	Lose	It is challenging and enjoyable
**Subject 2**	Lose	Win	win	Lose	Lose	The sphere has too much speed
**Subject 3**	Win	Lose	Win	Lose	Lose	The route should be extended
**Subject 4**	Lose	Win	Lose	Lose	Lose	It is a difficult game to control with the armband
